# Connectivity‐Guided Individualized iTBS of the Posterior Parietal Cortex in Enduring Anorexia Nervosa: Network‐Level Target Engagement and Multimodal Response in a Single Case

**DOI:** 10.1002/cns.71206

**Published:** 2026-10-10

**Authors:** Enrico Collantoni, Sofia Gentili, Arianna Menardi, Antonino Vallesi, Giovanni Librizzi, Elena Tenconi, Andrea Serino, Gaia Risso, Daniele Olivo, Fabio Sambataro, Angela Favaro, Valentina Meregalli

**Affiliations:** ^1^ Department of Neurosciences University of Padua Padova Italy; ^2^ Padua Neuroscience Center University of Padua Padova Italy; ^3^ MySpace Lab Lausanne University Hospital, University of Lausanne Lausanne Switzerland; ^4^ NeuroRehab Research Center Lausanne University Hospital, Institution of Lavigny Lavigny Switzerland; ^5^ Institute of Health, School of Health Sciences HES‐SO Valais‐Wallis Sion Switzerland

**Keywords:** body representation, case report, eating disorder, individualized targeting, neuromodulation, parietal cortex, TMS

## Abstract

**Aims:**

Anorexia nervosa (AN) is a severe, often enduring disorder with scarce biological treatments, largely unable to address its core implicit features: habit‐like food restriction and body‐experience disturbances. We report a single‐case application of resting‐state connectivity‐guided intermittent theta‐burst stimulation (iTBS) to the posterior parietal cortex (PPC), a hub for body representation, peripersonal space, and affordance‐based action.

**Methods:**

A 31‐year‐old woman with 12 years of treatment‐resistant restricting AN, enrolled in an ongoing sham‐controlled feasibility trial, underwent 20 active iTBS sessions to an individualized left‐PPC site maximally coupled with a body‐representation × affordance reference network, assessed at baseline, post‐treatment, and four‐month follow‐up.

**Results:**

The protocol was fully feasible and well tolerated, without significant adverse events. Clinical change was gradual, with most of the weight gain occurring after the stimulation period: BMI rose from 15.65 to 16.01 at four‐month follow‐up and 16.40 over subsequent clinical follow‐up. Blinded global improvement was minimal; anxiety fell below threshold, whereas eating‐disorder and body‐shape scores remained at floor. Target–reference connectivity increased in the a priori direction, toward the healthy‐control mean. Exploratory measures changed heterogeneously, only partly tracking engagement.

**Conclusion:**

This case supports the feasibility and mechanistic plausibility of individualized parietal targeting in enduring AN, motivating a controlled trial to test its efficacy.

**Trial Registration:**

The parent trial is registered on ClinicalTrials.gov (NCT07106645)

## Graphical abstract

A 31‐year‐old woman with 12 years of treatment‐resistant anorexia nervosa received 20 sessions of resting‐state connectivity‐guided iTBS individually targeted to the posterior parietal cortex, showing increased BMI, strengthened target‐network connectivity, and changes in core implicit symptoms: body‐experience disturbance and food approach‐avoidance tendencies.

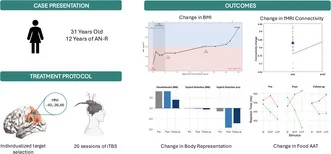

## 1 Introduction

Anorexia nervosa (AN) is a severe psychiatric disorder characterized by persistent food intake restriction, intense fear of weight gain, and a profound disturbance in the experience of one's own body [1]. Its course is frequently protracted, and long‐duration presentations respond poorly to available treatments [2, 3]. Biological interventions remain notably scarce: no pharmacological treatment has regulatory approval, and options for treatment‐resistant AN are extremely limited [4], motivating the development of neurobiologically grounded approaches. We recently developed a protocol of intermittent theta‐burst stimulation (iTBS) targeting the posterior parietal cortex (PPC) [5], aimed at two dimensions central to long‐duration AN and particularly resistant to treatment: the rigid, habit‐like maintenance of caloric restriction and the disturbance of body experience. The PPC's relevance to the latter is well established: it integrates multisensory signals to construct and update bodily self‐representations and to code peripersonal space, the interface defining the boundary between body and environment [6, 7]. It is further implicated in affordance coding and the transformation of perceived object properties into goal‐directed actions [8, 9], a mechanism directly relevant to the former, as food‐related affordances may drive automatic approach or avoidance responses sustaining caloric restriction. Consistent with this, we recently showed that repeated TMS over the intraparietal sulcus modulates implicit approach‐avoidance tendencies toward food in healthy individuals [10], suggesting the PPC as a candidate target for the implicit, habit‐like components of food‐related behavior. Neuromodulation in AN has so far primarily targeted the dorsolateral or dorsomedial prefrontal cortex, with preliminary evidence of efficacy [11, 12, 13]. A first limitation is that these approaches engage frontal circuits subserving higher‐order cognitive control and emotion regulation, leaving mechanisms more directly tied to disturbed body experience and habit‐like restriction comparatively unaddressed. A second limitation is the reliance on fixed anatomical coordinates, which ignores the individual variability in functional network organization known to influence stimulation efficacy [14, 15]. Our protocol addresses both issues through individualized targeting: for each participant, stimulation coordinates are derived from resting‐state functional magnetic resonance imaging (fMRI) as the PPC region, among a preselected pool, showing maximal connectivity with a reference network involved in body representation and affordance processing [5]. Here we report a single‐case application in a participant with long‐standing, treatment‐resistant restricting‐type AN enrolled in an ongoing feasibility trial. We report, in order of evidential priority: (i) feasibility, safety, and tolerability; (ii) clinical course, indexed by body weight, blinded global clinical impression, and self‐report; (iii) network‐level target engagement, operationalized as an a priori increase in resting‐state connectivity between the individualized PPC target and its defining body‐representation × affordance network; and (iv) an exploratory multimodal characterization spanning neuropsychological functioning, implicit and explicit body representation, peripersonal space, and implicit food‐related action tendencies. The case is intended to establish feasibility and mechanistic plausibility, not clinical efficacy.

## 2 Methods

### 2.1 Case Presentation

A 31‐year‐old female patient with restricting‐type AN according to DSM‐5‐TR criteria was enrolled in a TMS‐based feasibility trial at the Eating Disorders Unit of the University Hospital of Padova (see next paragraph for details). The patient had an illness duration of approximately 12 years, with onset at age 19 years. Prior treatment included multiple outpatient interventions and one semi‐residential rehabilitation program, none of which had achieved sustained clinical remission. She therefore met the trial inclusion criterion of adequate illness duration and at least one prior unsuccessful treatment. No psychotropic medication was prescribed at baseline or during the study period. No psychiatric or medical comorbidities were documented. Concomitant treatment‐as‐usual throughout the stimulation period consisted of standard outpatient clinical follow‐up at the Eating Disorders Unit, with no treatment intensification during the trial.

### 2.2 Treatment Protocol

The protocol, described in detail in [10] was approved by the Ethics Committee of the Hospital of Padova (protocol 6168/EST/25) and registered on ClinicalTrials.gov (NCT07106645); the patient provided written informed consent for participation and publication of case‐level data. Structural T1‐weighted and resting‐state fMRI were acquired three days before the first session. The stimulation target was derived from the patient's rs‐fMRI. A reference network was defined meta‐analytically through Neurosynth by intersecting two thresholded maps, one for implicit behavioral tendencies (“affordance”, “habit”, “motor program”) and one for bodily self‐representation (“body representation”, “body schema”, “peripersonal”, “multisensory integration”, “embodiment”), retaining only voxels surviving a 2‐SD threshold in both domains. From a bilateral pool of 37 posterior parietal parcels of the Schaefer‐200 atlas [16], the target was defined as the parcel maximally functionally connected with this network. For this patient, it corresponded to area 35 in the left PPC, centered at MNI [−40, −36, 48]. Coil positioning was guided by frameless stereotaxic neuronavigation on the patient's T1. Stimulation comprised 20 iTBS sessions over 20 consecutive weekdays, delivered with a MagPro system and a Cool B70 A/P active/sham coil (MagVenture, Farum, Denmark). Each session delivered 600 pulses in a standard iTBS pattern (three‐pulse 50‐Hz bursts at 200‐ms intervals, 2‐s on/8‐s off). iTBS was chosen because it increases cortical excitability comparably to conventional high‐frequency rTMS [17] but in markedly shorter sessions, improving feasibility and tolerability for repeated daily administration. Intensity was set at 100% of the individual resting motor threshold, estimated at Session 1 with the Stochastic Approximation Motor Threshold procedure (SAMT) [18] and re‐evaluated at the first session of each subsequent week (Sessions 6, 11, 16).

### 2.3 Outcome Measures

Assessments were administered at three time points: baseline (T0), the week immediately following the final stimulation session (T1), and 4 months after baseline (T2). MRI was acquired only at T0 and T1. Body weight was additionally recorded at routine outpatient visits beyond T2; these observations lie outside the protocol assessment window and are reported as clinical follow‐up. A complete description of all outcome measures can be found in [5]. This report focuses on the outcomes below.

#### 2.3.1 Safety and Tolerability

At the end of each session, the patient rated the discomfort experienced during stimulation on a 0–100 scale and reported any side effect or adverse event occurring since the previous session.

#### 2.3.2 Clinical Course

The clinical course was indexed by two first‐order outcomes. Body mass index (BMI) was calculated at each time point and at routine outpatient visits across the study period. Global illness severity and change were rated by a blinded, non‐treating psychiatrist with no prior knowledge of the patient using the Clinical Global Impression scale (CGI). The severity item (CGI‐S; 1 = normal, not ill, to 7 = among the most extremely ill) was scored at T0 and T1 with reference to the eating‐disorder population and to the patient's state over the preceding week; the improvement item (CGI‐I; 1 = very much improved, 4 = no change, 7 = very much worse) was scored at T1 relative to T0 by the same rater.

At each time point the patient also completed the Eating Disorder Examination Questionnaire (EDE‐Q) [19], the Body Shape Questionnaire (BSQ‐34) [20], and the State–Trait Anxiety Inventory (STAI) [21], assessing eating disorder psychopathology, body image disturbance, and anxiety symptoms, respectively.

A nine‐item visual analogue scale (VAS; 0–100) was completed before each of the 20 stimulation sessions, assessing desire to eat, urge to restrict food intake, meal‐related anxiety, body comfort, body dissatisfaction, body‐focused attention, urge for physical activity, cognitive flexibility, and anxiety. Session‐by‐session changes were examined with two complementary approaches in R, using the scan package [22]. Linear trend analysis yielded an unstandardized slope coefficient (B), representing the average change in VAS score per session. The Non‐overlap of All Pairs (NAP) [23] then compared Week 1 (Sessions 1–5) with Week 4 (Sessions 16–20). NAP is a non‐parametric effect size expressing the percentage of between‐phase pairs in which the later score exceeds the earlier one: 50% indicates no effect, values above 65% indicate a medium effect, and values above 73% indicate a large effect [23]. Statistical significance was assessed via the Wilcoxon rank‐sum test (W), with the direction of expected change specified a priori for each item.

#### 2.3.3 Target Engagement (Resting‐State Functional Connectivity)

Details on the neuroimaging acquisition and preprocessing steps are available in [10]. In brief, structural images underwent brain extraction, bias field correction, tissue segmentation and MNI normalization by means of a combination of ANTs (Advanced Normalization Tools) [24] and FMRIB Software Library (FSL)'s algorithms [25]. Functional MRI data underwent correction for motion‐related and scanner‐derived artifacts (bias field) using FSL and ANTs' functions, respectively [24, 26]. Artifact Rejection Toolbox (ART) procedures and Components‐based Noise Correction (CompCor) [27] were applied to remove of outlier scans (scrubbing) and removal of white matter and cerebrospinal fluid principal components, as per the CONN denoising pipeline [28]. Finally, coregistration of each participant's functional and anatomical volume was carried out via FMRIB's Linear Image Registration Tool (FLIRT) [29] and smoothing was applied with a Gaussian kernel with a full width at half maximum (FWHM) of 6 mm. Subject‐space functional data were parcellated into 200 regions of interest (ROIs) according to the Schaefer atlas [16] and regional time series were extracted from both the atlas ROIs and the Neurosynth‐derived networks' overlap map, i.e., the body × affordance reference network.

To assess if the iTBS protocol significantly modulated the brain resting state connectivity patterns, the post‐treatment connectivity between the individualized PPC target and the body × affordance reference network was computed via Pearson's correlation and compared to the one assessed at baseline. The patient's pre‐ and post‐treatment connectivity values were contextualized against the distribution of values obtained from a reference sample of healthy female participants (*n* = 44, mean age = 24.93 ± 3.57, mean BMI = 20.90 ± 1.71) scanned with the same MRI protocol and acquisition sequences in the context of a separate study.

#### 2.3.4 Multimodal Characterization

##### 2.3.4.1 Neuropsychological Functioning

Executive function and cognitive flexibility were assessed with the Wisconsin Card Sorting Test (WCST) [30], and central coherence with the copy trial of the Rey Complex Figure Test, from which the central coherence index was calculated (RCFT) [31, 32].

##### 2.3.4.2 Body Representation

Explicit body representation was assessed with a figure rating scale of computer‐generated silhouettes spanning BMI 12–38, from which the patient selected the silhouettes matching her perceived and desired body. Two indices were derived: body size distortion (perceived minus actual BMI) and body dissatisfaction (desired minus perceived BMI).

Implicit body representation was assessed with a virtual‐reality (VR) task in which a green dot approached the patient from varying orientations and heights; she was instructed to press a button at the exact moment she perceived the stimulus to have “touched” her body. This yields a perceptual map of body boundaries in the absence of visual or tactile feedback, from which a distortion index was derived (perceived minus actual boundary, in cm; negative values indicate underestimation).

##### 2.3.4.3 Peripersonal Space

Peripersonal space representation was assessed with a VR visuo‐tactile task adapted from an established paradigm [7]: while a visual stimulus approached the patient in VR, a tactile stimulus was delivered at one of five possible distances, and she responded as quickly as possible. Because tactile responses are facilitated once the approaching stimulus enters peripersonal space, the reaction‐time profile across distances provides an implicit estimate of its extent (boundary). The task was administered separately with high‐calorie food and neutral‐object stimuli. For each distance, a facilitation index was computed as bimodal (visuo‐tactile) minus unimodal (tactile‐only) reaction times, with more negative values indicating greater facilitation. Facilitation indices were fitted with a linear function across distances, yielding an intercept reflecting the overall extent of the PPS boundary and a slope reflecting the sharpness of the transition between peripersonal and extrapersonal space.

##### 2.3.4.4 Implicit Food Attitudes

Implicit behavioral tendencies toward food were measured with a mobile version of the Approach‐Avoidance Task ([5, 33]), in which the patient pulled or pushed a smartphone in response to images of high‐calorie food, low‐calorie food, and neutral objects; approach bias was computed as the mean reaction‐time difference between push and pull movements.

## 3 Results

### 3.1 Safety and Tolerability

The patient completed all 20 stimulation sessions without interruption. Discomfort ratings were 0 for the majority of sessions, with a maximum value of 20/100 reported on Sessions 8, 9, and 16. Regarding side effects, the patient reported a mild headache on a single occasion (Session 7), which appeared after the end of stimulation and resolved spontaneously within approximately 1 h without the use of medication. No other adverse events were reported. The patient consistently reported a positive subjective experience and high overall tolerability.

### 3.2 Clinical Course

The patient remained underweight throughout but gained weight steadily across the study period (Figure 1). Body weight rose from 42.60 kg at baseline (BMI 15.65, DSM‐5‐TR “severe” range) to 43.20 kg at the end of stimulation (BMI 15.87) and 43.60 kg at the four‐month follow‐up (BMI 16.01), crossing into the “moderate” range. Within the stimulation period the increase was confined to the first week (+0.70 kg), after which weight plateaued until T1. It then rose slowly to T2 (+0.40 kg) and continued to accrue over routine clinical follow‐up, reaching 44.65 kg (BMI 16.40) at 28 weeks: a total gain of 2.05 kg, or 0.75 BMI units, over six and a half months (Figure 1).

**FIGURE 1:**
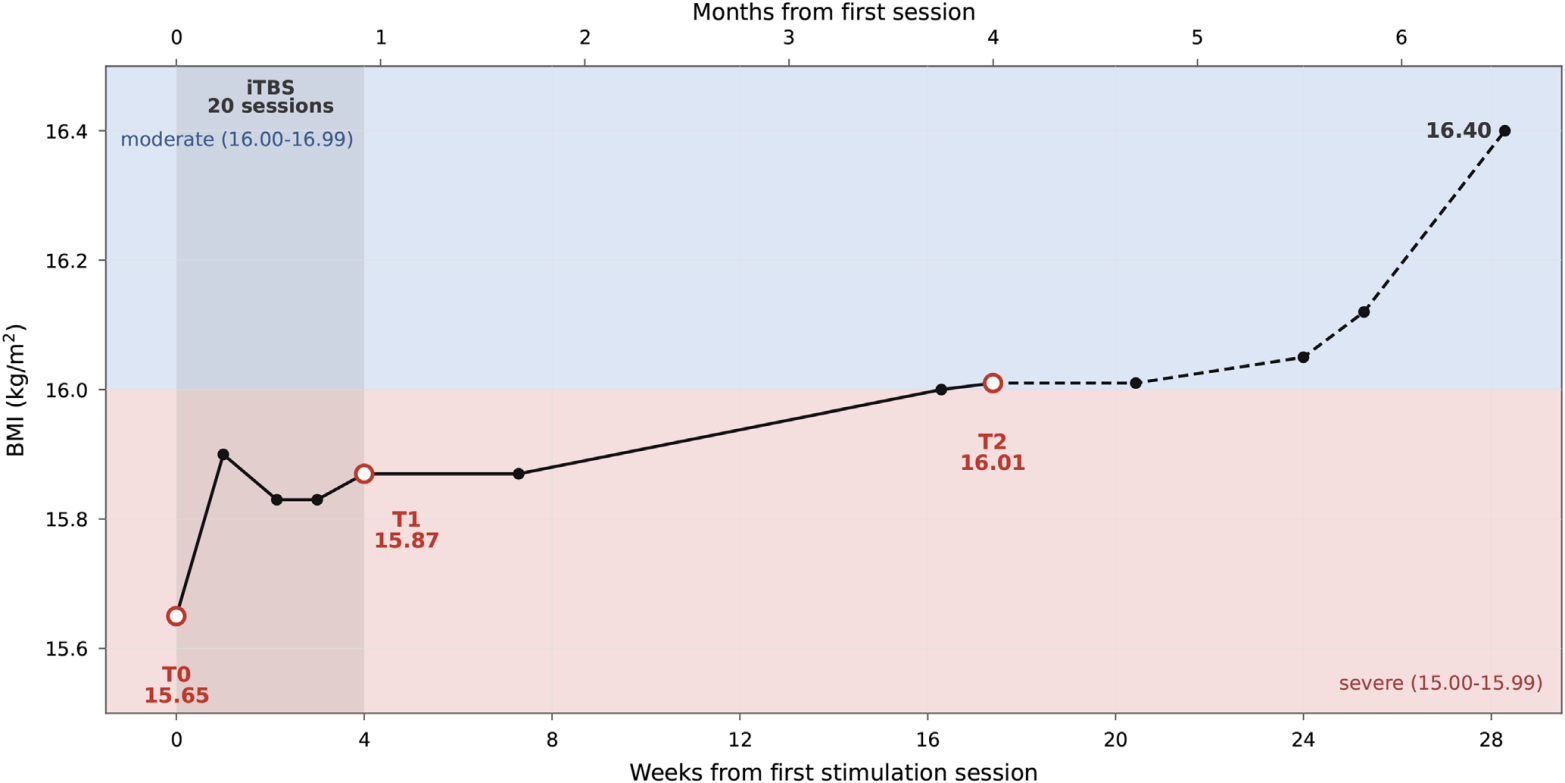
BMI trajectory. Body mass index (kg/m^2^) against weeks from the first stimulation session. Shaded band, four‐week stimulation period; horizontal bands, DSM‐5‐TR severity ranges. Open markers, protocol time points (T0 baseline, T1 post‐treatment, T2 four‐month follow‐up) at their scheduled positions; filled markers, routine outpatient visits; dashed segment, clinical follow‐up beyond the protocol window.

On the blinded CGI, illness severity was rated moderately ill (CGI‐S = 4) at both baseline and post‐treatment, with a global improvement rating of minimally improved (CGI‐I = 3) at post‐treatment relative to baseline. Beyond the blinded assessment, over the follow‐up period, the treating team observed increased behavioral flexibility and initiative in managing everyday demands, consistent with improved global functioning. This observation was unblinded.

As regards self‐report measures, the patient showed a reduction in both state and trait anxiety across the protocol. At baseline, scores on both STAI subscales were at the clinically relevant cutoff (state = 40, trait = 44) [34], decreasing to 31 and 39, respectively at follow‐up, falling below the threshold for clinically significant anxiety. Eating‐disorder psychopathology (EDE‐Q) showed a slight increase from baseline (0.39) to follow‐up (0.42), remaining well below the clinical cutoff throughout [35]. Similarly, body‐shape concerns (BSQ) showed a negligible increase from 38 to 40, with scores at all time points remaining far below the threshold for clinical concern [20]. These self‐report scores were thus at floor throughout. Session‐by‐session VAS changes are reported in Table 1 and Figure 2. Comfort in one's own body, body dissatisfaction, and desire to eat improved consistently across the protocol, showing trends in the expected direction and the three largest NAP values in the dataset; comfort in one's own body reached a statistically significant Week 1–Week 4 difference. Anxiety and need to exercise showed large negative trends but modest NAP values, driven by a markedly elevated first‐session score followed by near‐zero scores thereafter, suggesting initial pre‐session agitation rather than progressive improvement. Urge to restrict, meal‐related anxiety, and body focus showed negligible trends, remaining close to zero throughout, while flexibility showed a modest negative trend.

**TABLE 1:** Session‐by‐session changes in VAS scores.

Measure	W1 (M)	W2 (M)	W3 (M)	W4 (M)	B	NAP	W	*p*
Desire to eat	1.2	1.2	4.4	4.2	0.226	76	6.0	0.097
Urge to restrict	0.0	0.0	0.0	0.2	0.010	40	10.0	0.885
Meal anxiety	0.0	0.0	1.0	0.4	0.046	30	7.5	0.951
Body comfort	51.0	56.0	52.0	68.0	0.914	**100**	0.0	0.**004**
Body dissatisfaction	42.0	32.0	38.0	36.0	−0.226	72	18.0	0.128
Body focus	2.2	0.6	1.4	2.8	0.035	20	5.0	0.957
Physical activity	4.0	0.0	0.0	0.0	−0.286	60	15.0	0.212
Flexibility	44.0	42.0	42.0	40.0	−0.195	34	16.5	0.859
Anxiety	11.2	0.6	0.4	1.2	−0.717	64	16.0	0.250

*Note:* M W1–W4, mean VAS score per week; B, unstandardized linear trend coefficient; NAP, Non‐overlap of All Pairs; W, Wilcoxon rank‐sum statistic; *p* = two‐tailed *p*‐value. Bold values indicate statistical significance (*p* < 0.05).

**FIGURE 2:**
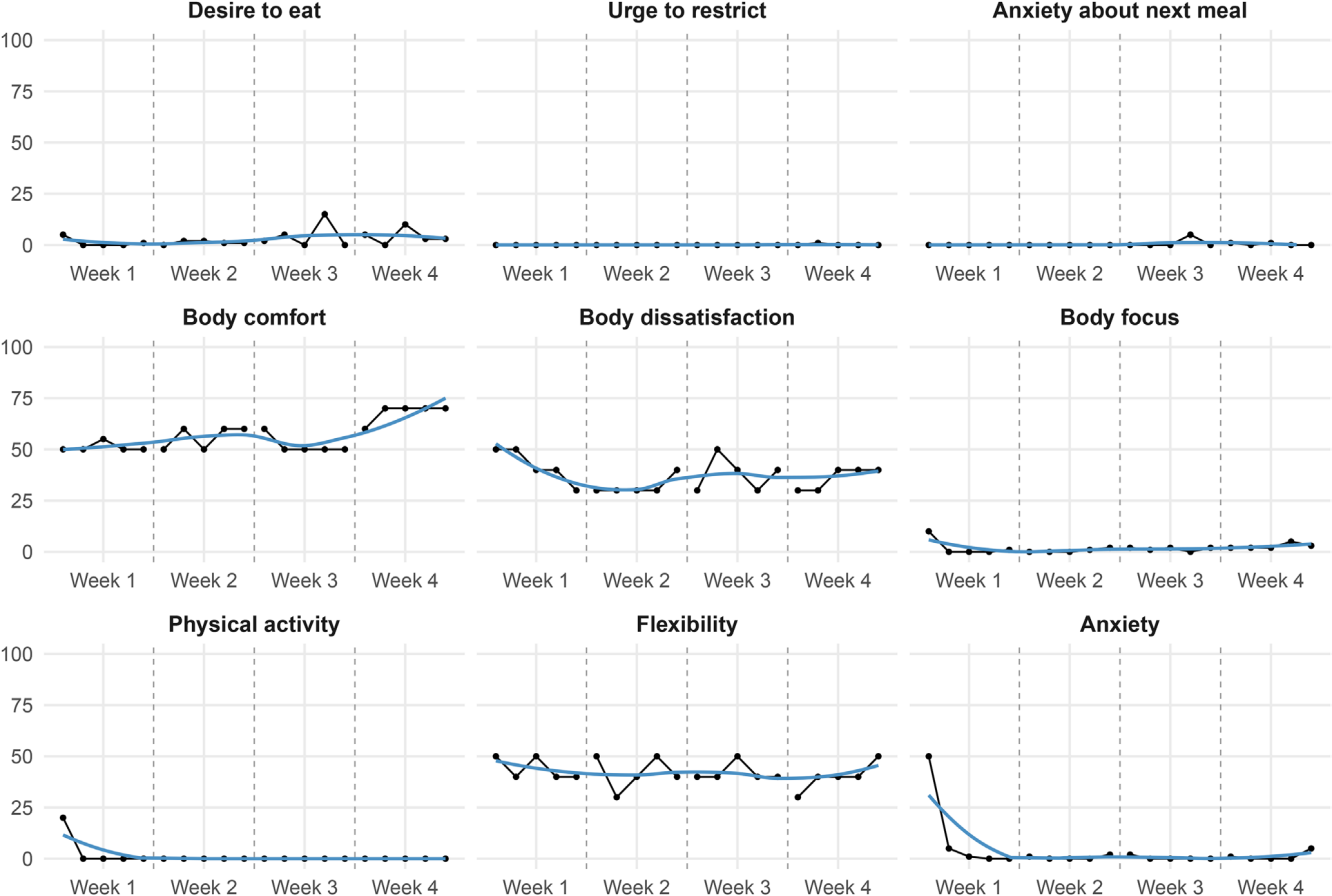
Daily self‐reported outcome measures across the 20 iTBS sessions (Weeks 1–4). Each panel displays a distinct outcome domain, rated on a 0–100 scale. Black lines represent raw session‐by‐session scores; blue lines represent the moving average across sessions.

### 3.3 Target Engagement (Resting‐State Functional Connectivity)

At baseline, connectivity between the individualized PPC target (MNI [−40, −36, 48]) and the body × affordance reference network was *r* = 0.76, *p* < 0.0001. After 20 iTBS sessions, connectivity increased to *r* = 0.84, *p* < 0.0001, shifting the patient toward the control‐group mean. The change was in the a priori hypothesized direction, consistent with facilitatory iTBS strengthening coupling within the targeted network (Figure 3).

**FIGURE 3:**
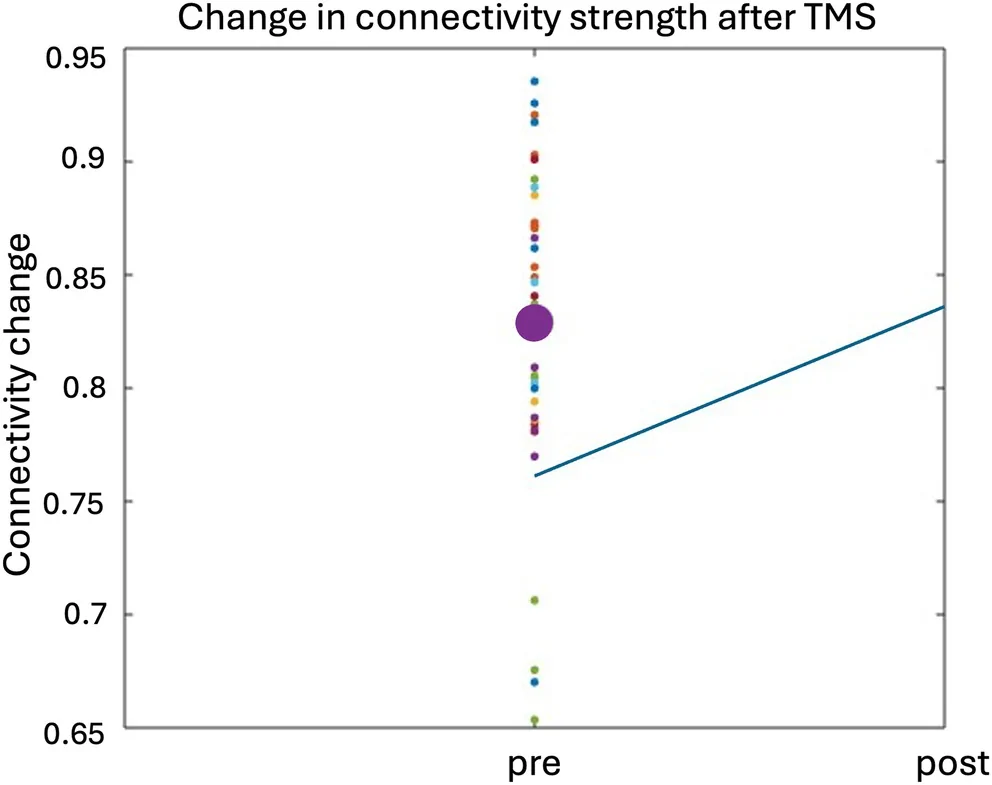
Target engagement. The pre‐ and post‐intervention changes in connectivity between the TMS target in the PCC and the body × affordance reference network are shown. At baseline, the participant showed weak connectivity between the two regions, which strengthened over time (blue line). Following iTBS, the participant's connectivity reached a value comparable to the average (purple marker) observed in a control group of *n* = 44 healthy individuals. Each individual correlation value in the control group is represented by a uniquely colored dot.

### 3.4 Multimodal Characterization

#### 3.4.1 Neuropsychological Profile

Performance on the WCST improved markedly across assessments. At baseline, the patient showed a severely impaired executive profile (51% correct responses; 31 perseverative errors and 36 perseverative responses; 1/6 categories completed), at the 1st percentile relative to normative data [30]. At post‐treatment, performance improved substantially and globally (80% correct; 8 perseverative errors and responses; all six categories completed; 50th percentile), improving further at follow‐up (84% correct; 5 perseverative errors and responses; 70th percentile).

Central coherence remained within a good range across all assessment points (1.67, 1.64, and 1.47 at baseline, post‐treatment, and follow‐up, respectively), with a slight decrease observed at follow‐up.

#### 3.4.2 Body Representation

On the figure rating scale, the patient reported at all three time points a desired BMI higher than her perceived body size, indicating a consistent wish to gain weight. A reduction in dissatisfaction of two BMI units was observed at follow‐up (4, 4, and 2 BMI units at baseline, post‐treatment, and follow‐up, respectively). Explicit body size distortion was minimal at baseline (−0.01 BMI units), with a slight increase in underestimation at post‐treatment and follow‐up (−0.09 and −0.30 BMI units, respectively).

A similar pattern of increasing underestimation emerged from the implicit body representation task. At baseline, the patient showed minimal distortion (−0.73 cm), which increased at post‐treatment and follow‐up (−4.60 and −3.54 cm, respectively) (see Figure 4).

**FIGURE 4:**
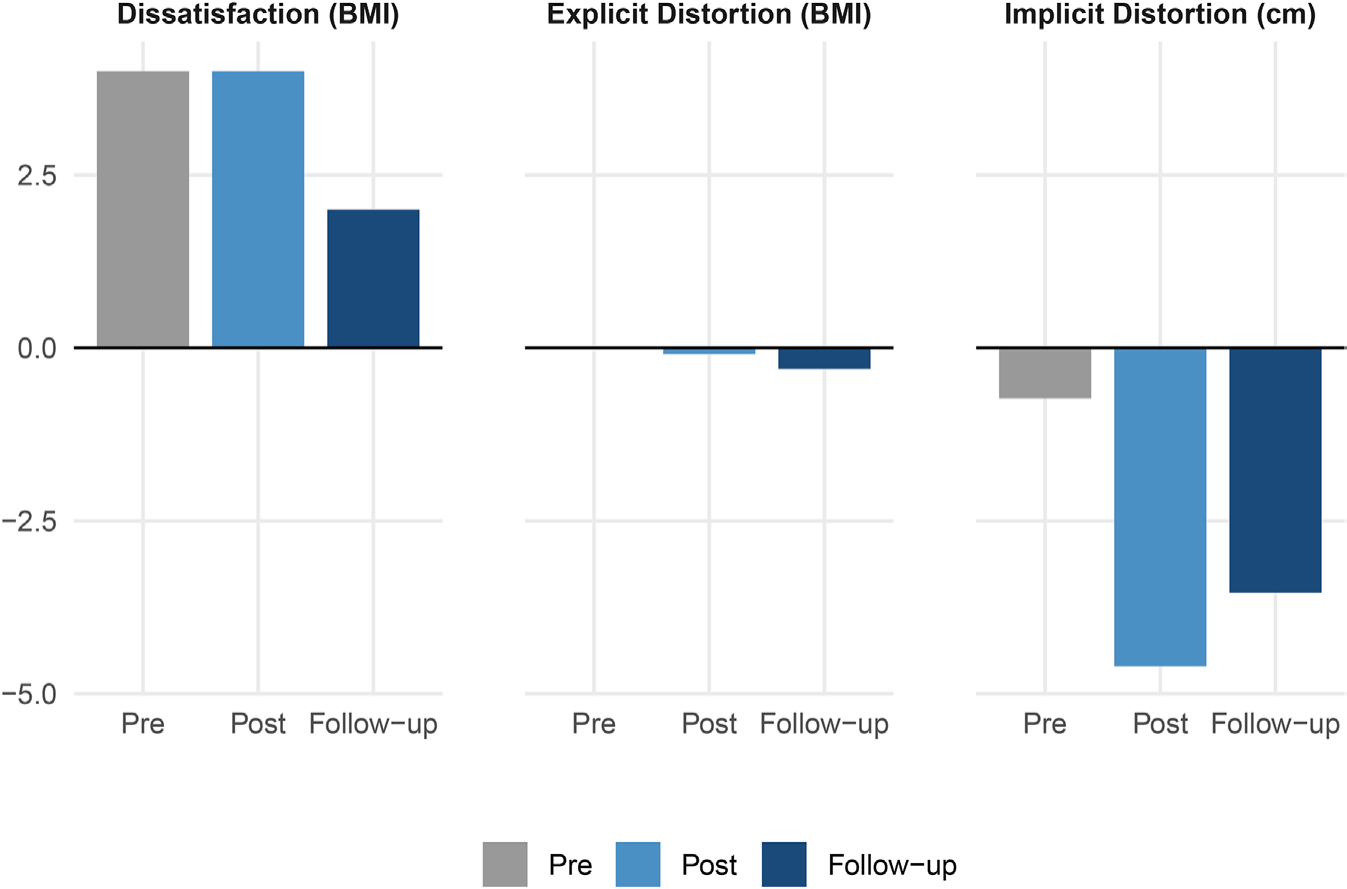
Body representation indices at pre‐treatment, post‐treatment, and follow‐up assessments. Left panel: Body dissatisfaction, calculated as desired minus perceived BMI. Middle panel: Explicit body distortion, calculated as perceived minus actual BMI. Right panel: Implicit body distortion, calculated as perceived minus actual body boundary (in cm).

#### 3.4.3 Peripersonal Space

In the Peripersonal Space task, the patient showed a marked change between baseline and post‐treatment, which remained stable at follow‐up (Figure 5a). This change was not specific to food stimuli and was primarily reflected in the extension of the PPS, as indicated by the intercept for both food and object stimuli, which shifted from −55.6/−57.0 at baseline to −1.23/−7.59 at post‐treatment, indicating a larger PPS extension before stimulation.

**FIGURE 5:**
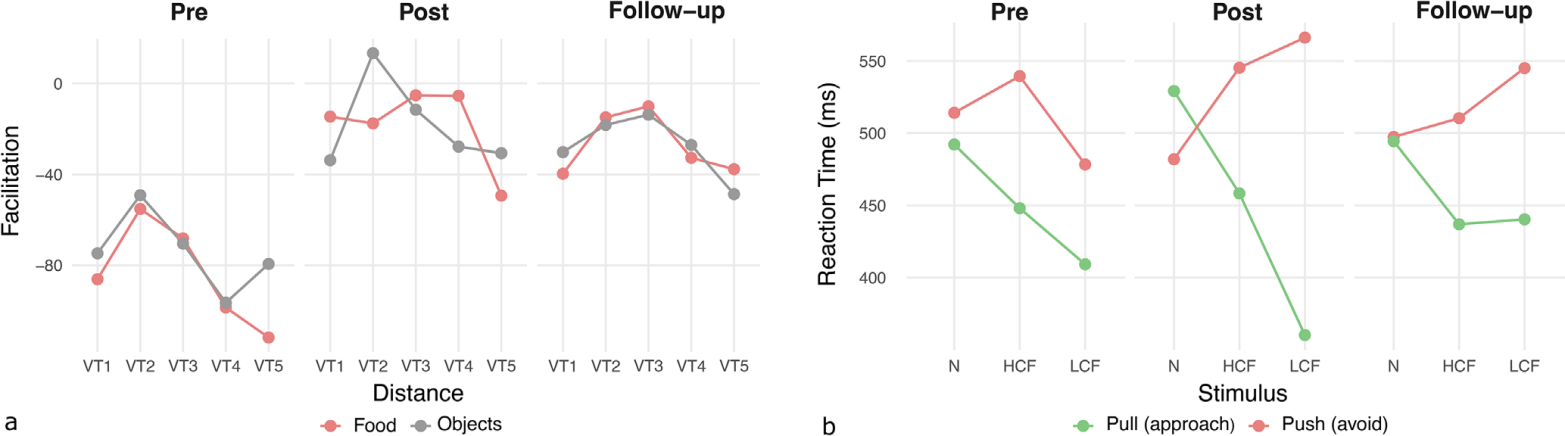
(a) Facilitation index across the five visuo‐tactile distances (VT1‐VT5), from farthest to nearest at pre‐treatment, post‐treatment, and follow‐up, separately for food and neutral‐object stimuli; (b) reaction times in the Approach‐Avoidance Task as a function of stimulus type (N, neutral; HCF, high‐calorie food; LCF, low‐calorie food) and response direction (pull/approach vs. push/avoid), shown separately for pre‐treatment, post‐treatment, and follow‐up.

#### 3.4.4 Implicit Food Attitudes

In the Approach‐Avoidance Task, the patient showed a positive approach bias at baseline for both high‐calorie (91.4 ms) and low‐calorie food stimuli (69.2 ms), with a smaller bias for neutral objects (22.0 ms). The largest change across the protocol was observed for low‐calorie food stimuli, for which the approach bias increased markedly at post‐treatment (205.9 ms) before returning toward baseline levels at follow‐up (104.7 ms). The approach bias for high‐calorie food stimuli showed a gradual decrease across the three assessment points (91.4, 87.0, and 73.4 ms, respectively) (see Figure 5b).

## 4 Discussion

This case provides the first individual‐level demonstration of an individualized, resting‐state connectivity‐guided iTBS protocol targeting the PPC in enduring, treatment‐resistant AN. The protocol was fully feasible and uniformly well tolerated, with all 20 sessions completed, no significant adverse effects, and a consistently positive subjective experience. On clinically anchored outcomes, the change was modest but sustained: she remained underweight throughout, yet her BMI crossed from the DSM‐5‐TR severe into the moderate range at follow up, and continued to rise thereafter, while the blinded CGI indicated minimal improvement. Although modest in absolute terms, this trajectory is noteworthy in the context of the patient's long‐standing illness and history of unsuccessful treatments; its clinical significance and its relationship to the intervention, however, cannot be established from this single uncontrolled case, for three reasons in particular. First, most of the total weight gain occurred after the protocol window under continued treatment‐as‐usual; such delayed, cumulative change is compatible with the LTP‐like plasticity iTBS is thought to induce [36] but, in an uncontrolled single case, cannot be dissociated from natural course or non‐specific factors. Second, within the stimulation period itself, weight increase was confined to the first week and did not continue, a time course an uncontrolled design cannot attribute to the intervention. Moreover, weight increase was not accompanied by improvements in eating disorder psychopathology, so that convergence across different dimensions of the disorder was lacking, although, as discussed below, this measure was already below the cutoff at baseline. Third, the gains in behavioral flexibility, initiative, and global functioning noted by the treating team were unblinded and are reported as clinical observation. A convergent but similarly qualified pattern emerged on the WCST, where cognitive flexibility improved from the 1st to the 70th percentile: consistent with the observed clinical gains, but not fully dissociable from practice effects. Self‐reported anxiety (STAI) fell from a clinically relevant to a subthreshold level, whereas eating‐disorder and body‐shape scores remained below clinical cut‐offs throughout and were essentially unchanged. This floor is itself consistent with the enduring, largely egosyntonic phenotype that motivated the protocol, in which restriction operates as an entrenched behavioral automatism rather than as actively distressing weight‐related cognition. Repeated VAS measurement nonetheless revealed a gradual improvement in the patient's relationship with her body, with increased body comfort and reduced body dissatisfaction. Against this background, the mechanistic readout moved in the predicted direction: connectivity between the individualized PPC target and the body‐representation × affordance reference network increased following stimulation, consistent with the a priori hypothesis and the excitatory nature of iTBS, reaching a value comparable to the healthy‐control average. Within the constraints of a single case, the observed change in connectivity was in the expected direction, consistent with network‐level target engagement. This result should nonetheless be interpreted with caution, given the inherent test–retest variability of resting‐state acquisitions. Future trials could account for such fluctuations through dynamic connectivity analyses or repeated longitudinal scans.

All the measures most directly tied to parietal function changed across the protocol. Both implicit and explicit body‐size representation shifted toward greater underestimation across the protocol. Although superficially interpretable as a deterioration, this direction resembles the bias typical of healthy controls, who tend to underestimate their body size—a bias associated with more positive body‐related attitudes [37]. The peripersonal‐space intercept increased markedly from baseline to post‐stimulation: the strongly negative baseline facilitation, even at the farthest positions, indicates a PPS larger than typically reported in healthy controls [38], and appeared to normalize after stimulation. Notably, this change emerged for both food and object stimuli, consistent with a general effect on the multisensory body‐boundary representation supported by the PPC rather than a food‐specific mechanism. Implicit food‐related action tendencies were also modulated across time points. Taken together, these changes are compatible with the hypothesized neural engagement and mechanistically plausible as downstream signatures of parietal modulation, given the PPC's role in coding body metrics, peripersonal space, and affordance‐based action ([7, 10, 39]). Their direction was heterogeneous, however, and a single case cannot establish whether they relate to the observed connectivity increase.

The study's principal strength is conceptual and methodological rather than clinical. It deploys a broad assessment combining resting‐state connectivity with virtual‐reality and mobile behavioral paradigms, neurocognitive testing, peripersonal‐space measures, and session‐by‐session self‐report, characterizing changes at network, cognitive, perceptual, implicit‐behavioral, and subjective levels within a single case. Further strengths include the a priori specification of the target‐engagement hypothesis, normative referencing of the connectivity outcome via the Crawford–Howell method, blinded psychiatric assessment, and a structured timeline with four‐month follow‐up.

The limitations are those intrinsic to a single case: no causal or efficacy inference is warranted, since the absence of a control condition, the open‐label context, and concurrent treatment‐as‐usual preclude attributing any change to stimulation. This extends to the clinical follow‐up data reported beyond the protocol window, which were collected under routine outpatient care and are equally susceptible to confounding by concurrent treatment and by the natural course of illness. As a single case in a young woman with a specific illness profile and duration, the findings are not generalizable to AN more broadly, and should be read as supporting feasibility and mechanistic plausibility in this instance rather than as generalizable evidence of efficacy. The connectivity outcome rests on single pre‐ and post‐treatment acquisitions, so the observed shift cannot be distinguished from the test–retest variability of the metric, which we could not estimate in this individual; the engagement claim should therefore be read as directionally consistent with the hypothesis rather than as an established effect.

## 5 Conclusion

Within these constraints, the case supports a narrow but useful conclusion. An individualized connectivity‐guided parietal iTBS protocol is deliverable and tolerable in enduring AN, and network‐level target engagement in the predicted direction was observed in this case; the accompanying clinical improvement was modest, and the behavioral sequelae were heterogeneous. These observations motivate the ongoing sham‐controlled trial (NCT07106645), designed to test whether parietal target engagement translates into clinically meaningful change, to incorporate test–retest characterization of the connectivity metric and control‐network specificity, and to adopt outcomes sensitive to the low‐distress profile of long‐duration AN.

## Author Contributions

Enrico Collantoni: conceptualization; methodology; investigation; funding acquisition; writing – original draft; writing – review and editing. Valentina Meregalli: conceptualization; methodology; investigation; writing – original draft; writing – review and editing. Sofia Gentili: conceptualization; methodology; investigation; writing – review and editing. Arianna Menardi: conceptualization; methodology; formal analysis; writing – review and editing. Antonino Vallesi: conceptualization; methodology; formal analysis; writing – review and editing. Giovanni Librizzi: investigation; writing – review and editing. Daniele Olivo: formal analysis; writing – review and editing. Andrea Serino: methodology; writing – review and editing. Gaia Risso: methodology; writing – review and editing. Elena Tenconi: methodology; writing – review and editing. Fabio Sambataro: supervision; writing – review and editing. Angela Favaro: supervision; writing – review and editing. All authors read and approved the final version of the manuscript.

## Funding

This work was supported by the STARS@UniPD 2023 program of the University of Padova.

## Ethics Statement

The study was approved by the Ethics Committee of the Hospital of Padova (protocol 6168/EST/25) and was conducted in accordance with the ethical standards laid down in the Declaration of Helsinki.

## Consent

The patient provided written informed consent for participation in the study and for the publication of case‐level data.

## Conflicts of Interest

The authors declare no conflicts of interest.

## Data Availability

The data that support the findings of this study are available on request from the corresponding author. The data are not publicly available due to privacy or ethical restrictions.
